# Telomere length and epigenetic age acceleration in adolescents with anxiety disorders

**DOI:** 10.1038/s41598-021-87045-w

**Published:** 2021-04-08

**Authors:** Angelica Cerveira de Baumont, Mauricio Scopel Hoffmann, Andressa Bortoluzzi, Gabriel R. Fries, Patrícia Lavandoski, Lucas K. Grun, Luciano S. P. Guimarães, Fátima T. C. R. Guma, Giovanni Abrahão Salum, Florencia M. Barbé-Tuana, Gisele G. Manfro

**Affiliations:** 1 Anxiety Disorders Outpatient Program for Children and Adolescents, Protaia, Federal University of Rio Grande do Sul, UFRGS/Hospital de Clínicas de Porto Alegre, HCPA, Porto Alegre, Brazil; 2 Graduate Program in Psychiatry and Behavioral Sciences, Federal University of Rio Grande do Sul, UFRGS, Porto Alegre, Brazil; 3 Graduate Program in Neuroscience, Institute of Basic Sciences/Health, Federal University of Rio Grande do Sul, UFRGS, Porto Alegre, Brazil; 4 Basic Research and Advanced Investigations in Neurosciences, BRAIN Laboratory, Hospital de Clínicas de Porto Alegre, HCPA, Porto Alegre, Brazil; 5 Translational Psychiatry Program, Department of Psychiatry and Behavioral Sciences, McGovern Medical School, University of Texas Health Science Center at Houston, (UTHealth), Houston, TX USA; 6 Graduate Program in Biochemistry, Laboratoy of Molecular Biology and Bioinformatics, Federal University of Rio Grande do Sul, UFRGS, Porto Alegre, Brazil; 7 Unit of Epidemiology and Biostatistics, Hospital de Clínicas de Porto Alegre, HCPA, Porto Alegre, Brazil; 8 Departamento de Neuropsiquiatria, Universidade Federal de Santa Maria, Avenida Roraima 1000, Santa Maria, 97105-900 Brazil; 9 Care Policy and Evaluation Centre, London School of Economics and Political Science, London, UK; 10 Instituto Nacional de Psiquiatria do Desenvolvimento para Crianças e Adolescentes (INPD), Conselho Nacional de Desenvolvimento Científico e Tecnológico (CNPq), Porto Alegre, RS Brazil; 11 Group of Inflammation and Cellular Senescence, Graduate Program in Cellular and Molecular Biology, School of Sciences, Pontifícia Universidade Católica do Rio Grande do Sul (PUCRS), Porto Alegre, RS Brazil; 12 Postgraduate Program in Pediatrics and Child Health, School of Medicine, Pontifical Catholic University of Rio Grande do Sul (PUCRS), Porto Alegre, Brazil; 13 Serviço de Psiquiatria, Hospital de Clínicas de Porto Alegre, HCPA, Rua Ramiro Barcelos, 2350-sala 400N, Rio Branco, Porto Alegre, RS 90035-903 Brazil

**Keywords:** Biomarkers, Medical research, Molecular medicine, Development of the nervous system, Epigenetics in the nervous system

## Abstract

Evidence on the relationship between genetics and mental health are flourishing. However, few studies are evaluating early biomarkers that might link genes, environment, and psychopathology. We aimed to study telomere length (TL) and epigenetic age acceleration (AA) in a cohort of adolescents with and without anxiety disorders (N = 234). We evaluated a representative subsample of participants at baseline and after 5 years (n = 76) and categorized them according to their anxiety disorder diagnosis at both time points: (1) control group (no anxiety disorder, n = 18), (2) variable group (anxiety disorder in one evaluation, n = 38), and (3) persistent group (anxiety disorder at both time points, n = 20). We assessed relative mean TL by real-time quantitative PCR and DNA methylation by Infinium HumanMethylation450 BeadChip. We calculated AA using the Horvath age estimation algorithm and analyzed differences among groups using generalized linear mixed models. The persistent group of anxiety disorder did not change TL over time (p = 0.495). The variable group had higher baseline TL (p = 0.003) but no accelerated TL erosion in comparison to the non-anxiety control group (p = 0.053). Furthermore, there were no differences in AA among groups over time. Our findings suggest that adolescents with chronic anxiety did not change telomere length over time, which could be related to a delay in neuronal development in this period of life.

## Introduction

Anxiety disorders are characterized by maladaptive responses to threats and are considered one of the largest group of disabling mental disorders in childhood and adolescence^1^. The estimated lifetime prevalence of anxiety disorders in studies with children or adolescents is about 15–20%^2^ and is associated with a considerable degree of fluctuation in diagnostic status over time^3,4^. The persistence of anxiety symptoms and the chronicity of anxiety disorders are common, with recurrence of the same or different diagnoses across several time points throughout life^5–7^.

From a physiological perspective, anxiety disorders are stressful conditions, normally accompanied by deregulation of stress systems such as the hypothalamic–pituitary–adrenal axis, the autonomic nervous system, and the immune system^8,9^. The presence of different stressful conditions has been associated with accelerated aging^10–12^ evaluated through telomere length (TL) shortening and epigenetic age acceleration (AA) from DNA methylation (DNAm) profiles of cytosine phosphate guanines (CpGs). These measurements can be used as markers of cellular age according to different studies^12–15^. Despite the association between stress, immune system and anxiety, and between stress and age acceleration, few studies have addressed TL and AA as biomarkers in anxiety disorders.

TL is considered a “biological clock” but the literature has shown conflicting results when evaluating the relationship between leukocyte TL as a biological aging marker and psychiatric disorders^16^. Some studies reported that TL is associated with the vulnerability to anxiety disorders^17–19^, however other prospective studies evaluating if anxiety precedes telomere shortening showed inconsistent findings^20–23^. Two recent meta-analyses have shown small (− 0.06)^24^ to moderate effect sizes (− 0.53)^16^ in telomere shortening in adult patients with an anxiety disorder.

The recent literature has suggested AA as an estimate of the discrepancy between DNAm age and chronological age, measurable by residual from a regression model of DNAm age against chronological age^14,25–28^. Some studies investigated the association between DNAm age with posttraumatic stress disorder^29,30^, schizophrenia^31,32^, depression^33^, generalized anxiety disorder^29^ and bipolar disorder^26^, and also found inconsistent findings. In adolescence, AA was associated with inflammation, the probability of middle age cardiovascular disease^34^, as well as higher odds for internalizing and thought problems^35^.

Although markers of cellular aging have been studied in psychiatric disorders^16,33,36,37^, there are few studies evaluating this association in youth^23,35^. Therefore, it is important to understand if a prevalent and early onset disorder such as anxiety disorder could accelerate aging at the molecular level and what would be the implications for neuronal development in this sensitive phase. Thus, to investigate if anxiety disorder would modify the cellular aging markers in adolescents, we longitudinally evaluated the association between TL, AA, and anxiety disorders over 5 years in this age group.

## Results

### Baseline characteristics

The sample re-evaluated after 5 years (n = 76) was representative of the whole 234 non-medicated children and adolescents recruited from public schools at baseline as described elsewhere^38^. Similarly, the sub-sample we used to evaluate the epigenetic data did not differ from the whole sample according to Bortoluzzi et al.^39^. Descriptive data about the baseline sample and the 5-year follow-up one is depicted in Table 1. The mean age of the total sample at baseline was 13 years of age, 61% was female and most of the participants have Caucasian ancestry.

**Table 1:** Descriptive data on adolescent anxiety diagnostic evaluated in baseline and follow-up.

Variables	Baseline				5-years follow-up			
	Typical development (n = 18)	Variable (n = 38)	Persistent (n = 20)	Total sampleª (N = 76)	Typical development (n = 18)	Variable (n = 38)	Persistent (n = 20)	Total sampleª (N = 76)
**Telomere length (base pairs)**								
Mean (SD)	1.22 (0.566)	2.20 (1.47)*	1.25 (0.826)	1.70 (1.23)	0.759 (0.359)**	0.853 (0.628)**	1.13 (0.528)	0.891 (0.553)**
**Epigenetic age acceleration (years)**								
Mean (SD)	− 1.45 (7.98)	0.119 (8.07)	− 3.03 (3.86)	− 1.14 (7.21)	2.05 (4.14)	0.582 (3.82)	1.86 (4.51)**	1.33 (4.06)**
**Age (years)**								
Mean (SD)	13.2 (2.42)	13.8 (2.48)	12.5 (1.95)	13.4 (2.37)	17.2 (2.50)**	17.7 (2.58)**	16.3 (2.08)**	17.2 (2.48)**
**Sex**								
Female	9 (50%)	22 (58%)	15 (75%)	46 (61%)				
Male	9 (50%)	16 (42%)	5 (25%)	30 (39%)				
**Race/ethnicity**								
Caucasian	10 (56%)	25 (66%)	13 (65%)	48 (63%)				
African Brazilian	2 (11%)	5 (13%)	4 (20%)	11 (14%)				
Mixed	5 (28%)	8 (21%)	3 (15%)	16 (21%)				

Chi-squared test was applied for testing sex and race/ethnicity between anxiety groups. Analysis of variance and Tukey post-hoc tests were used to examine telomere length, epigenetic age acceleration and chronological age differences among anxiety groups within each year and for overall sample between years (a). *p < 0.05 compared with all other anxiety groups within the same time point (Tukey post-hoc test); **p < 0.05 compared with the same group at baseline (t test).

### TL and AA within and between the two time points

We considered as outcomes, in our analyses, the telomere length (TL) and the epigenetic age acceleration (AA) defined as the residuals from regressing DNA methylation (DNAm) age on chronological age. We assessed the mean between-group differences at each time point and within-group differences (and for the overall sample) across the time points (Table 1). The TL of the variable group was significantly higher as compared with the control and with the persistent group at baseline (F_(2,47)_ = 4.212, p = 0.021). At the second evaluation, there were no TL differences between the anxiety groups. There were no differences in AA among the groups at both evaluations. Cellular aging markers have different mean levels from baseline to follow-up within each anxiety group and in the overall sample. The TL of the variable group decreased from the first to the second assessment (t_(36)_ = 4.10, p = 3.16 × 10^–3^), which was not observed for AA (t_(34)_ =  − 0.237, p = 0.818). The same effect was observed for the persistent group regarding TL (t_(16)_ = 2.45, p = 0.025), and AA (t_(16)_ =  − 1.454, p = 0.162). However, the TL of the persistent group did not change over time (t_(18)_ = 0.467, p = 0.645), whereas AA increased (t_(18)_ =  − 2.728, p = 0.013). The TL of the overall sample decreased from the first to the second assessment (t_(74)_ = 4.21, p = 8.13 × 10^–5^) and AA increased (t_(74)_ =  − 2.009, p = 0.048). Data are shown in Table 1 using ANOVA and Tuckey post-hoc tests for between-group comparisons and t test for within-group comparisons.

We also analyzed possible correlations between TL and AA at baseline and follow-up using Spearman correlations and found that TL did not correlate with AA either at baseline (r = 0.194; p = 0.305) or at follow-up (r = 0.182; p = 0.287). The correlation of chronological age with TL (r = − 0.302, p = 0.002) and with DNAm age (r = 0.283, p = 0.007) were significant. Nonetheless, the correlation between AA with chronological age was not significant (r = -0.015; p = 0.903).

### TL and AA changes across anxiety disorder groups over time

**Figure 1:**
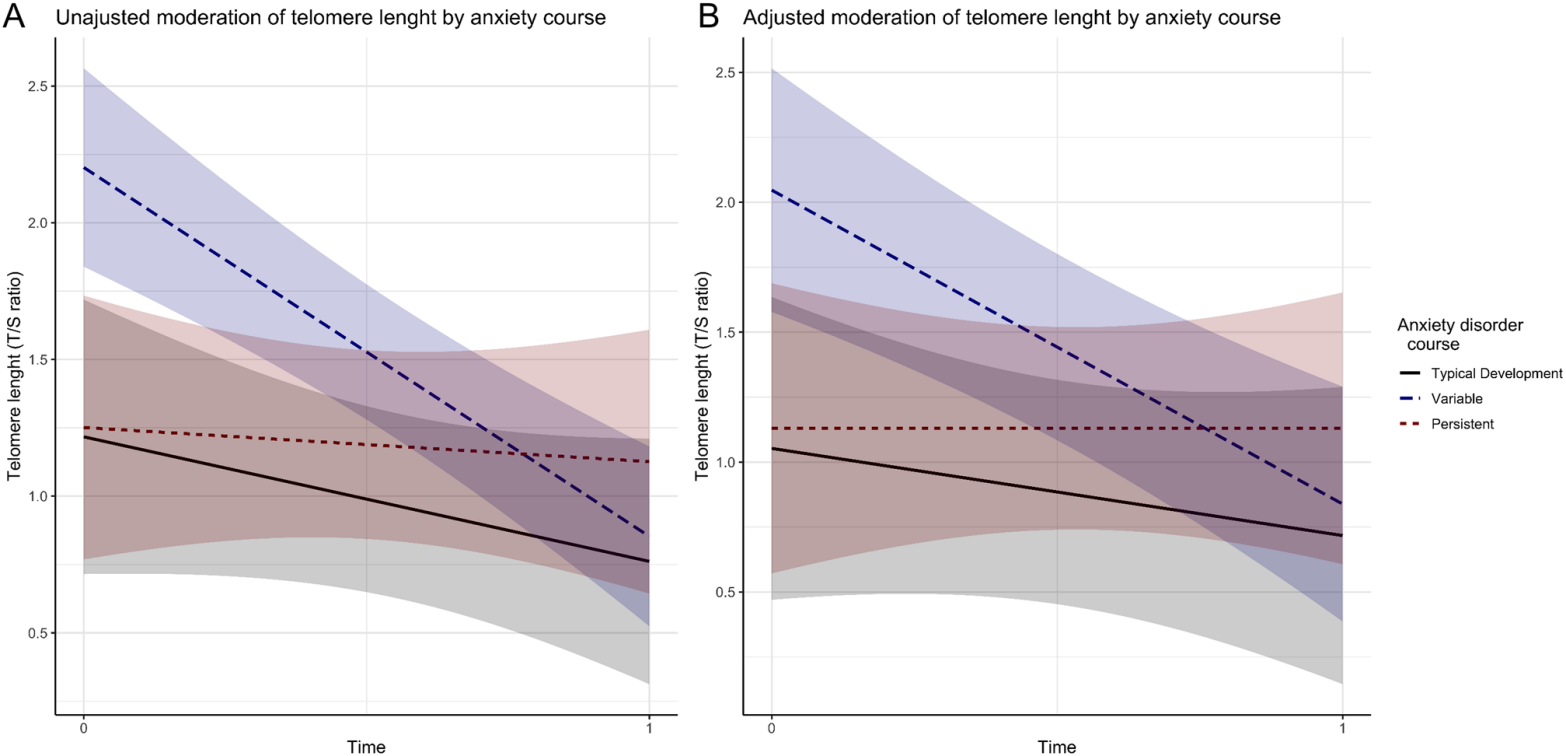
Unadjusted (**A**) and adjusted (**B**) interaction of anxiety diagnostic course (colored lines with shaded 95% CI) on telomere length change (y-axis) between baseline and follow-up five years later (x-axis).

**Figure 2:**
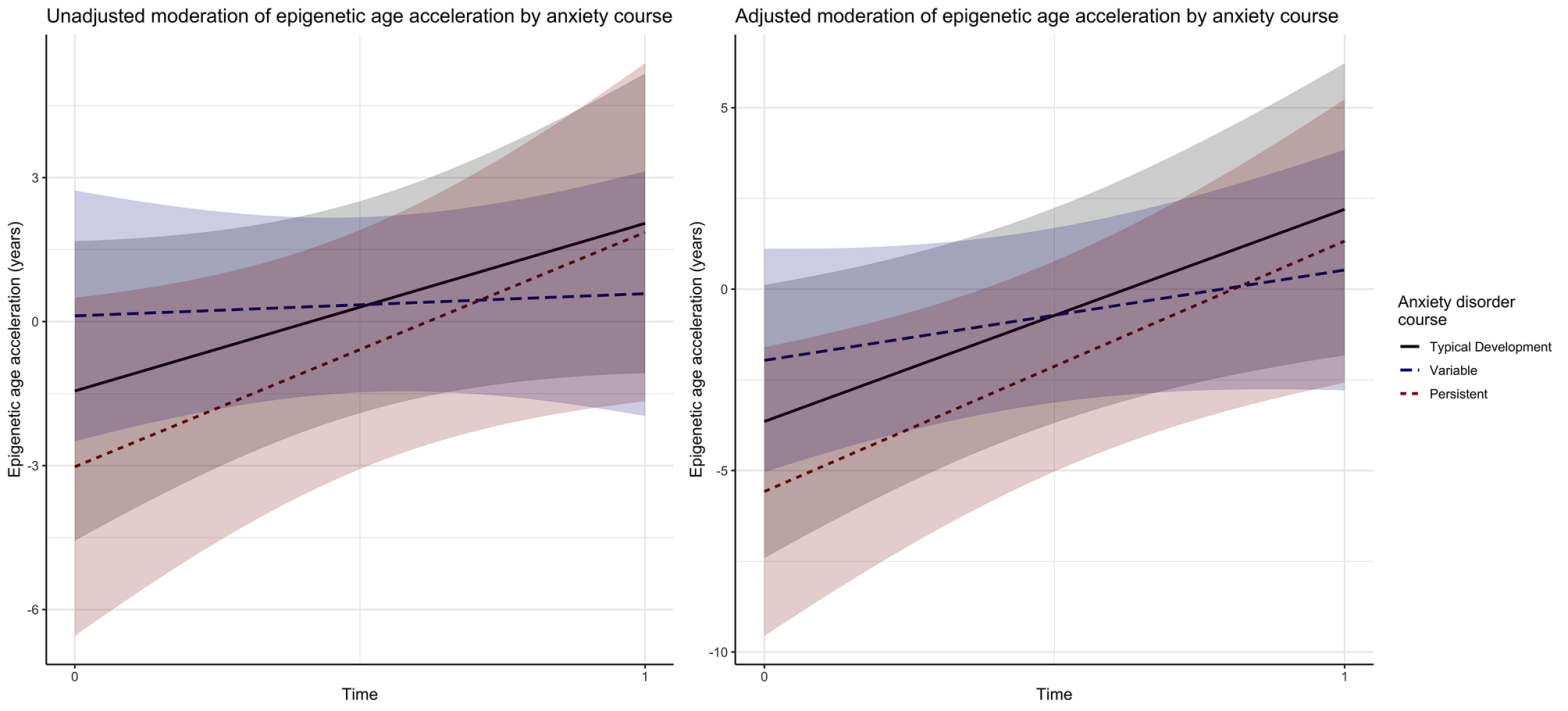
Unadjusted (**A**) and adjusted (**B**) interaction of anxiety diagnostic course (colored lines with shaded 95% CI) on epigenetic age acceleration change (y-axis) between baseline and follow-up five years later (x-axis).

We conducted a generalized linear mixed models (GLMM) analysis to examine whether TL and AA changed across anxiety disorder groups. Considering the TL, our data suggested no significant difference in the persistent group compared to control (p = 0.834 in the adjusted model) at baseline. However, we found a significant difference in the variable group, which had higher baseline TL in comparison to the control group (T/S ratio = 0.989; 95% CI 0.384, 1.594; p = 0.003 in the adjusted model, Fig. 1; Table S1). Furthermore, considering TL changes over time, we also found that the persistent group did not change its TL over time (p = 0.495 for the group by time interaction in the adjusted model). Moreover, we demonstrated that the variable group had higher TL erosion rate in comparison to control (T/S ratio = − 0.894; 95% CI − 1.711, − 0.076; p = 0.038), nevertheless when we adjusted the model for age, sex and ethnicity, this difference was no longer significant (T/S ratio = − 0.864; 95% CI − 1.696, − 0.031; p = 0.053, Fig. 1; Table S1). There were no significant differences among the groups regarding the AA (Fig. 2; Table S2).

We decomposed the variable group into the incident and the remittent anxiety courses and run the same set of analysis as a sensitivity analysis (see “Supplementary information”). Descriptive data of these groups can be found in Table S3. Results of the GLMM analysis demonstrated that both the incident and the remittent groups have the same associations with higher TL as reported by the variable group and no association with AA when compared to controls, revealing the consistency of combining incident and remittent anxiety courses into one group only (Figures S1, S2; Tables S4, S5). We also performed two supplementary linear regression models to predict TL and AA changes over time (deltas) by baseline anxiety diagnostic status to further understand the association of anxiety with cellular aging markers without considering data structure and anxiety courses. Considering the linear model, we found no association of anxiety diagnostic status versus no diagnosis at baseline with TL and AA changes (see “Supplementary information”).

## Discussion

In this study, we aimed to investigate how cell aging markers could be associated with anxiety disorder over time in a sample of adolescents. For this propose, we investigated relative TL shortening and AA as markers of cellular aging, in a community sample of adolescents in two different evaluations, at baseline and after 5 years. Different from our a priori hypothesis, the persistent group of anxiety disorder had neither shortened TL nor higher AA in comparison to controls. Instead, this group interestingly did not change telomere length over time. On the other hand, the variable group had higher baseline TL but no accelerated TL erosion in comparison to the control group, after adjusting for age, sex and ethnicity. These findings suggest that although the general trend was TL shortening over time, individuals with persistent chronic anxiety disorders did not follow the trend of shortening the TL.

TL generally decreases progressively over the lifespan. In early life stages, telomeres undergo shortening at an accelerated rate of approximately 170–270 bp per year, which is gradually reduced until reaching a plateau^40,41^, ranging between 32.2 and 45.5 bp per year in adult studies^36,42^. In this sense, we hypothesized if the lack of TL shortening found in the persistent group could be associated with a delay in brain maturation in chronic anxious adolescents. Some studies suggested that elevated anxiety and depression might be characterized by a delayed maturation of the ventromedial prefrontal cortex and an altered trajectory of cortical thinning^43,44^. Similarly, there has been debate as to whether attention-deficit hyperactivity disorder (ADHD) is a consequence partly of delay in brain maturation, particularly in prefrontal regions related to attention and motor planning^45^.

On the other hand, a variable or non-persistent course of anxiety disorders seems to be less deleterious than a persistent course. A recent multigenerational study showed clinically relevant differences among children without anxiety or with low anxiety, non-persistent anxiety, and persistent anxiety in both generations^46^. Therefore individuals with remittent or incident (variable) anxiety course seem to be different from those with a chronic anxiety disorder. It is important to note that higher severity of symptoms and clinical severity indicators have been associated with higher persistence of anxiety disorders^5,6,47^. Thus, we can suggest that adolescents with a non-persistent (variable) course of anxiety disorders would have less severe symptoms and that they have a developmental trajectory closer to those from the typical developmental course. In agreement, Newman et al.^43^ hypothesized that, in children with higher generalized anxiety, an increase in VMPFC “territory” may be somewhat delayed as compared to those without anxiety.

Telomere length and telomerase activity seem to modulate neuronal differentiation since telomerase expression and activity is downregulated during neuronal differentiation^48^ and its overexpression appears to inhibit differentiation in neural cell lines^49^. Decline in telomerase activity during brain development signals cells to exit the cell cycle and to differentiate into neurons or glial cells^50^. Consistently, a study with rodents showed a marked decrease in telomerase activity throughout the mice brain during the early period of development^51^, and a recent study found increased TL associated to decreased brain weights, increased anxiety‐like behavior, altered pain response, and increased levels of inflammation in early life stress in adolescent rodents^52^.

As no participants from our sample were using psychiatric medication, our findings concerning no TL shortening in persistent anxiety could not be due to the use of pharmacotherapy with protective effect or elongation inducer of telomeres, such as lithium^53^. Nevertheless, we found a significantly higher TL baseline evaluation in the variable group, similar to the findings of the incident and remittent groups evaluated separately, even in age, gender, and ethnicity-controlled analyses. This could be due to genetic factors since TL had an estimated heritability of 0.70^54^.

Very few longitudinal studies have examined the association between anxiety disorder and TL. TL was shorter in individuals with remitted and current depressive and/or anxiety disorder (n = 2,292) as compared to healthy control subjects (n = 644) in a large longitudinal study^21^. However, these authors found no difference in telomere attrition rate among the groups when they evaluated TL and diagnosis status over time (from baseline to 6-year follow-up). Prospective studies testing the hypothesis that anxiety symptoms and diagnoses precede telomere shortening showed inconsistent findings^20–23,55^. Therefore, these inconsistent results and the lack of longitudinal studies reveal that there is a gap to be filled concerning TL and anxiety^56^. This is especially true if we considered young individuals.

Despite methodological limitations in telomere shortening studies, novel and more specific aging markers, such as an integrative analysis involving multiple biomarkers are needed. Thus, epigenetic aging can be used as an index of disproportionate or accelerated biological cell aging^57^. In our study, we found no significant differences in AA between anxiety disorder groups and controls in GLMM analysis. Wolf et al.^29^ examined if different psychiatric disorders, including generalized anxiety disorders (GAD), predicted acceleration of DNA methylation age over time, and failed to demonstrate any significant associations between this marker and GAD. Moreover, Fries et al.^26^ reported statistically differences in AA between bipolar patients and healthy controls only in a sub-sample of patients with more than 33-year-old. This data, in agreement with our study, suggested that epigenetic aging could not be a valuable marker of cellular aging or neural development in young individuals.

Furthermore, we were not able to demonstrate any correlations between TL and AA. Other studies investigating the correlation between these markers had also shown some contradictory results. Shorter TL was associated with increased extrinsic AA in postmenopausal women^15^, but telomere length did not significantly correlate with epigenetic accelerated aging in a study including bipolar disorder patients, their siblings, and healthy controls^26^. Although a mismatch can occur between DNAm age and chronological age^57^, studies suggested that AA and TL are uncorrelated. Both processes predict chronological age independently because epigenetic aging and cellular senescence are independent mechanisms that do not need to occur together^58,59^. Moreover, we were able to demonstrate correlation of chronological age with TL and DNAm age, but not with AA. Consistently, the correlation between Horvath DNAm age (predicted age) and chronological age is well described^27,30,57,60^. Nevertheless, since AA is all epigenetic age that is not explained by the chronological age, characterizing the acceleration, this variable does not necessarily correlate with chronological age, as shown in previous studies^30,61^.

Some limitations of the present study were the small sample size and the ethnic background of our population. However, we try to minimize this confounder, adjusting our analyses to ethnicity. Our small sample size may be underpowered to show possible differences among the groups. We attempt to minimize this by using a mixed-effect model, which uses all information available from both time points. Furthermore, only 47 subjects were selected to undergo genome-wide DNA methylation and AA estimation, which, despite small, was representative of the whole studied sample. Also, as we analyzed anxiety diagnoses as a group, we might have lost some specificity of the different anxiety disorders. Moreover, quantification of telomeres and DNA methylation analyses were derived from saliva samples which comprised heterogeneous cell types, however, several studies indicate that high-quality methylation profiles can be generated from saliva^62^. DNA methylation from saliva seems to be more similar to the DNA methylation from brain cells as compared to methylation in a blood cell sample^63^. Importantly, Horvath’s clock is a multi-tissue predictor of age that allows estimating the DNAm age and AA of most tissues and cell types, including saliva^57^. Similarly, studies demonstrated that telomere lengths from different tissues are significantly correlated^64–66^. Finally, some important factors associated with AA and/or TL, such as perceived stress, childhood adversities, educational attainment, body weight, physical activity, sleep duration, smoking, sex hormones, inflammation, and oxidative stress^67–69^ were not considered in this study.

On the other hand, our manuscript brings several new preliminary pieces of evidences about anxiety in childhood and adolescence. Our sample came from a middle income country that face different socioeconomic constraints, such as higher inequality, violence and precarious supportive network, important environmental factors associated with epigenetic factors. To our knowledge, no longitudinal studies were investigating epigenetic aging in adolescents using an extensive evaluation and following a stringent criterion for anxiety disorders diagnoses. Moreover, our study evaluated the association between anxiety disorders and two different biological clocks, using different and uncorrelated methodological procedures (TL and AA).

Therefore, our findings suggest that adolescents with persistent anxiety did not change telomere length over time, which could be associated with a delay in neuronal development in this period of life. Present findings also encourage TL as a more sensitive biomarker for anxiety disorders rather than AA. Further longitudinal studies evaluating these and other biological clocks over time in adolescence will allow us to better understand the cellular aging and associations with neuronal development, as well as the search for the best biomarkers in anxiety disorders at this life stage.

## Methods

### Sample selection and psychiatric evaluation

This study was approved by the Ethics Committee of the *Hospital de Clínicas de Porto Alegre* (HCPA), Brazil (protocol number 15–0349), and all parents or individuals over 18 years signed an informed consent form before entering the study. All procedures were followed in accordance with relevant guidelines.

Two hundred thirty four (n = 234) non-medicated children and adolescents were recruited from public schools in 2008 and assessed with an extensive psychiatric evaluation, as previously described by Salum et al.^3^. After these evaluations, 134 individuals were diagnosed with anxiety disorders and 100 individuals were classified as non-anxious controls. These 234 subjects were invited to participate in the 5-year follow-up survey and a total of 76 adolescents and young adults agreed to participate in this second phase of the study. They were re-evaluated using the Schedule for Affective Disorders and Schizophrenia for School-Age Children Present and Lifetime Version (K-SADS-PL) if less than 18 year-old or the Mini International Neuropsychiatric Interview (MINI)^70^ if 19 years of age or older. Saliva samples were collected in both evaluations to extract DNA.

We categorized the 76 participants that were evaluated twice into three groups according to their diagnostic status at both evaluations: (1) control group (no anxiety disorder at both time points, n = 18), (2) variable group (presence of any anxiety disorder in only one time point, n = 38) and (3) persistent group (anxiety disorder at both time points, n = 20). We further decomposed the variable group into incident (n = 16) and remittent groups (n = 22) and replicated all analysis considering these groups (see “Supplementary information”).

### DNA extraction

DNA was extracted from biological samples of saliva, collected at baseline (2008) and the end-point of follow-up (2013), using an Oragene Kit (DNA Genotek, Ottawa, Ontario, Canada). We checked nucleic acid concentration and purity spectrophotometrically (BioPhotometer Plus, Eppendorf, Hamburg, Germany) and all samples were stored at − 20 °C until subsequent analysis.

### Measurement of relative telomere length (T/S)

We used genomic DNA (25 ng/reaction) as template for quantification of relative mean TL (T/S) by real time quantitative polymerase chain reaction (qPCR)^71^, with minor modifications^60^. We performed two separate qPCR in the same position for each sample; one reaction amplified the telomere (T) repeated sequence while the other amplified a single copy gene, *36B4* (S), as a quantitative control. All reactions were performed in triplicate in separate 96-well plates. Relative TL was expressed as the T/S ratio for each participant. Primer sequences^71^ were (5′ → 3′): tel1, GGTTTTTGAGGGTGAGGGTGAGGGTGAGGGTGAGGGT; tel2, TCCCGACTATCCCTATCCCTATCCCTATCCCTATCCCTA and 36B4u, CAGCAAGTGGGAAGGTGTAATCC and 36B4d, CCCATTCTATCATCAACGGGTACAA. T and S master mix reactions followed the same concentrations with 0.1 × SYBR Green (Molecular Probes, CA, USA), 2 mM MgCl_2_, 0.1 mM each dNTP, 1% DMSO and 0.5 U of Platinum Taq DNA Polymerase (Invitrogen). Final primer concentrations for telomere amplification were 270 and 1,125 nM for 1 e 2 telomeres primers, respectively; and 300 and 500 nM for 36B4u and 36B4d primers, respectively. We performed PCR reactions in StepOnePlus Real-time PCR system (Applied Biosystems, CA, USA) and analyzed with StepOne Software v2.3 (Applied Biosystems). Amplification consisted of an initial incubation step for 2 min at 94 °C to activate hot start Platinum Taq DNA polymerase, followed by 22 cycles of denaturing at 94 °C for 15 s and annealing and extension for 2 min at 54 °C, for telomere amplification; and 30 cycles of denaturing at 94 °C for 15 s followed by annealing and extension for 2 min at 60 °C for *36B4* amplification. We confirmed the specificity of the amplification at the end of each run using melting curve analyses. Additionally, PCR products were confirmed using agarose gel electrophoresis. A reference sample was included in each run, as a calibrator to normalize the participants’ T/S ratio and calculate the final T/S ratio. At last, we generated standard curves for telomere and *36B4* amplification from the reference sample, over a fivefold range by serial dilution from 100 to 0.16 ng of gDNA to check for PCR amplification efficiency. Inter-plate variability was 2.7%.

### Array-based genome-wide DNA methylation assays

We extracted the genomic DNA (500 ng) from the saliva of the whole sample, however only 47 subjects paired by age, sex and different anxiety disorder trajectories due to logistical and financial limitations were included in the methylation analysis. The samples were treated with sodium bisulphite using the EZ-96 DNA Methylation-Gold Kit (Zymo Research, Orange, CA, USA) according to the manufacturer’s protocol. DNA methylation status was performed using the Infinium HumanMethylation450 (IHM450) BeadChip^72^ which covers 99% of Ref Seq genes regions and 96% of CpG islands/CpG island regions to explore the genome-wide DNA methylome^72–74^. Data regarding pre-processing of raw data of IHM450 BeadChip and differential methylation analysis can be found elsewhere^39^. All data of DNA methylation including methylated vs. unmethylated probes are deposited in GEO (GSE78975).

### Epigenetic age acceleration (AA)

DNA methylation age (DNAm age) and epigenetic age acceleration (AA) were calculated in samples using the Horvath age estimation algorithm^57^, which is a multi-tissue (including saliva) predictor of age that predicts DNAm age based on the methylation levels of 353 CpGs from the IHM450 BeadChip. As input to the age estimation algorithm, non-processed methylation data was used, as recommended by Horvath (https://horvath.genetics.ucla.edu/html/dnamage/faq.htm#_Toc385147415). This tool algorithm free available online (http://labs.genetics.ucla.edu/horvath/htdocs/dnamage/)^57^ provides an estimate of DNAm age and predicts AA (in years) using elastic net-penalized regression models (in R package) of DNAm age on chronological age. Regression models result in residuals that were compared among groups. Therefore, AA was defined as the residuals from regressing DNAm age on actual (chronological) age.

### Statistical analysis

Sample characteristics were described as median (minimum and maximum), means (standard deviations) or percentages. Chi-squared test was applied for testing sex and race/ethnicity between anxiety groups. We used analysis of variance (ANOVA) and Tukey post-hoc tests to examine TL, AA and chronological age differences among anxiety groups within each year. Furthermore, we used T test to examine mean differences in TL and AA for each anxiety group and for the overall sample between the two assessments.

We investigated the correlation between TL and AA, and correlations of TL, AA or DNAm age and chronological age using Spearman correlations due to the positive-skewness nature of these variables. We used generalized linear mixed models (GLMM) with random effects and adding interaction term with time, applying penalized quasi-likelihood parameter estimator to better fit non-normal distributed data^75^. This was calculated to examine if the anxiety groups are associated with the levels of TL or AA and their changes over time. We modeled TL and AA as dependent variables, time and anxiety disorder groups as fixed factors and subjects as random variable (random intercepts). To control for potential confounders, we adjusted the analysis for age, sex and ethnicity.

All statistical analyses were performed using SPSS v. 23.0 (SPSS Inc., Chicago, IL, USA) and in R, using the *MASS* package in R and the *glmPQL* function^76^. Significance levels were set to be p < 0.05.

### Ethics approval

This study was approved by the Ethics Committee of the Hospital de Clínicas de Porto Alegre (HCPA), Brazil (protocol number 15-0349). All procedures were followed in accordance with relevant guidelines.

### Consent to participate

All parents or individuals over 18 years signed an informed consent form before entering the study.

## Supplementary Information

Supplementary Information 1.

## Data Availability

All data of DNA methylation including methylated vs. unmethylated probes are deposited in GEO (GSE78975).
