# Supplementary material for: Telomere length and epigenetic age acceleration in adolescents with anxiety disorders
Source: Sci Rep. 2021 Apr 8;11:7716. doi: 10.1038/s41598-021-87045-w (PMC8032711; doi:10.1038/s41598-021-87045-w)
Supplement: Supplementary file 1 — Supplementary Information 1. [file 41598_2021_87045_MOESM1_ESM.docx]

**Telomere length and epigenetic age acceleration in adolescents with anxiety disorders**

Angelica Cerveira de Baumont^1,2,4,*^, Mauricio Scopel Hoffmann^8,9,2,10^, Andressa Bortoluzzi^1,3,4^, Gabriel R. Fries^5^, Patrícia Lavandoski^6^, Lucas K. Grun^12,11^, Luciano S. P. Guimarães^7^, Fátima T.C.R. Guma^6^, Giovanni Abrahão Salum^1,2,10^, Florencia M. Barbé-Tuana^6,11^, Gisele G. Manfro^1,2,3,4,10^

^1^ Anxiety Disorders Outpatient Program for Children and Adolescents, Protaia, Federal University of Rio Grande do Sul, UFRGS/Hospital de Clínicas de Porto Alegre, HCPA, Porto Alegre, Brazil.

^2^ Graduate Program in Psychiatry and Behavioral Sciences, Federal University of Rio Grande do Sul, UFRGS, Porto Alegre, Brazil.

^3^ Graduate Program in Neuroscience, Institute of Basic Sciences/Health, Federal University of Rio Grande do Sul, UFRGS, Porto Alegre, Brazil.

^4^ Basic Research and Advanced Investigations in Neurosciences, BRAIN Laboratory, Hospital de Clínicas de Porto Alegre, HCPA, Porto Alegre, Brazil.

^5^ Translational Psychiatry Program, Department of Psychiatry and Behavioral Sciences, McGovern Medical School, University of Texas Health Science Center at Houston, (UTHealth), Houston, TX, USA.

^6^ Graduate Program in Biochemistry, Laboratoy of Molecular Biology and Bioinformatics, Federal University of Rio Grande do Sul, UFRGS, Porto Alegre, Brazil.

^7^ Unit of Epidemiology and Biostatistics, Hospital de Clínicas de Porto Alegre, HCPA, Porto Alegre, Brazil

^8^ Universidade Federal de Santa Maria, Departamento de Neuropsiquiatria, Avenida Roraima 1000, Santa Maria, 97105-900, Brazil (UFSM).

^9^ Care Policy and Evaluation Centre, London School of Economics and Political Science, London, UK.

^10^ Instituto Nacional de Psiquiatria do Desenvolvimento para Crianças e Adolescentes (INPD), Conselho Nacional de Desenvolvimento Científico e Tecnológico (CNPq), Porto Alegre, RS, Brazil.

^11^ Group of Inflammation and Cellular Senescence, Graduate Program in Cellular and Molecular Biology, School of Sciences, Pontifícia Universidade Católica do Rio Grande do Sul (PUCRS), Porto Alegre (RS), Brazil.

^12^Postgraduate Program in Pediatrics and Child Health, School of Medicine, Pontifical Catholic University of Rio Grande do Sul (PUCRS), Porto Alegre, Brazil

***Corresponding author**:

**Angelica Cerveira de Baumont, MSc PhD**

Serviço de Psiquiatria, Hospital de Clínicas de Porto Alegre, HCPA

Rua Ramiro Barcelos, 2350 - sala 400N

Rio Branco, 90035-903 - Porto Alegre, RS - Brazil

Phone/Fax: +55 51 33598294

e-mail: angelbaumont@gmail.com

ORCID: 0000-0002-9477-8790

**Supplementary tables of Figures 1 and 2**

**Table S1.** Baseline and longitudinal analysis of relative telomere length in two different courses of anxiety disorders (variable and persistent) compared to TDC group, using generalized linear mixed regression models.

|  | Predictor | B^a^ | p | CI 95% |
| --- | --- | --- | --- | --- |
| Model 1^b^ | Variable AD | 0.985 ^**^ | 0.002 | 0.380 – 1.590 |
|  | Persistent AD | 0.034 | 0.923 | -0.646 – 0.714 |
|  | Time | -0.455 | 0.184 | -1.117 – 0.206 |
|  | Variable AD x Time | -0.894 ^*^ | 0.038 | -1.711 – -0.076 |
|  | Persistent AD x Time | 0.331 | 0.493 | -0.610 – 1.272 |
| Model 2^c^ | Variable AD | 0.989 ^**^ | 0.003 | 0.384 – 1.594 |
|  | Persistent AD | 0.076 | 0.834 | -0.614 – 0.766 |
|  | Time | -0.339 | 0.390 | -1.091 – 0.413 |
|  | Variable AD x Time | -0.864 | 0.053 | -1.696 – -0.031 |
|  | Persistent AD x Time | 0.339 | 0.495 | -0.612 – 1.291 |

Note: Negative values translate into shorter telomere length if compared to control. CI: Confidence Interval.

^a^ Telomere repeated sequence/single copy gene (T/S) ratio; ^b^ Non-adjusted; ^c^ Adjusted for age, sex and ethnicity; *p < 0.05; **p < 0.01.

**Table S2.** Baseline and longitudinal analysis of epigenetic age acceleration in two different groups of anxiety disorders (variable and persistent) compared to control group, using generalized linear mixed regression models.

|  | Predictor | B^a^ | p | CI 95% |
| --- | --- | --- | --- | --- |
| Model 1^b^ | Variable AD | 1.565 | 0.449 | -2.425 – 5.554 |
|  | Persistent AD | -1.580 | 0.508 | -6.192 – 3.033 |
|  | Time | 3.493 | 0.123 | -0.837 – 7.823 |
|  | Variable AD x Time | -3.031 | 0.299 | -8.648 – 2.587 |
|  | Persistent AD x Time | 1.393 | 0.679 | -5.135 – 7.920 |
| Model 2^c^ | Variable AD | 1.684 | 0.422 | -2.275 – 5.643 |
|  | Persistent AD | -1.931 | 0.426 | -6.500 – 2.638 |
|  | Time | 5.847 ^*^ | 0.027 | 1.006 – 10.688 |
|  | Variable AD x Time | -3.357 | 0.256 | -8.902 – 2.188 |
|  | Persistent AD x Time | 1.056 | 0.755 | -5.351 – 7.463 |

Note: Negative and positive values represent younger and older epigenetic ages compared to their chronological ages, respectively. CI: Confidence Interval. ^a^ Estimate of accelerated epigenetic aging (in years) by regressing DNAm age on chronological age; ^b^ Non-adjusted; ^c^ Adjusted for age, sex and ethnicity.*p < 0.05

***Supplementary analysis considering four anxiety groups***

We replicate the same analysis of the main text considering four anxiety groups (we categorized the variable group into two groups), namely (1) control group (no anxiety disorder at both time points, n=18), (2) incident group (presence of any anxiety disorder in the second time point but not in the first, n=16), (3) remittent group (presence of any anxiety disorder in the first time point but not in the second, n=22) and (4) persistent group (anxiety disorder at both time points, n=20). We reported this data in Table S3, and it is similar to Table 1 (main text) except for the incident and remittent groups.

***TL and AA within and between the two time points considering four anxiety groups***

We assessed the differences of TL and AA among the four above mentioned groups (Table S3). There were no TL differences between the anxiety groups at baseline (F_(3,44)_ = 2.75, p = 0.054) nor at the follow-up (F_(3,52)_ = 1.128, p = 0.347). The same applies for AA among the groups at baseline (F_(3,41)_ = 0.571, p = 0.637) and at follow-up (F_(3,42)_ = 0.452, p = 0.717). Cellular aging markers have different mean levels from baseline to follow-up within each anxiety group. Aside results presented in the main text for comparisons among controls and persistent groups across the two time points, the TL of the incident group decreased from the first to the second assessment (t_(14)_ = 2.656, p = 0.022), which was not observed for AA (t_(14)_ = 0.102, p = 0.921). The same was observed for the remittent group regarding TL (t_(20)_ = 3.059, p = 0.008), and AA (t_(20)_ = -0.589, p = 0.564).

***TL and AA changes across four anxiety disorder groups over time***

We used generalized linear mixed models (GLMM) analysis to examine whether TL and AA changed across these above mentioned four anxiety disorder groups. We found a significant difference in the incident and the remittent groups both with higher TL than the control group (Figure S1 and Table S4). However, considering TL changes over time, we also have not found an interaction of time with incident and remittent group in the unadjusted analysis, which was different when combining both groups into the variable group (see main text). There were no significant differences among the incident and remittent groups regarding the AA, as it was for the variable group described in the main text (Figure S2 and Table S5).

***TL and AA differences from baseline to follow-up predicted by baseline diagnostic status***

As a supplementary analysis, we have also estimated two linear regression models to examine if changes in TL and AA during the follow-up period could be predicted by baseline anxiety status (presence or absence of any anxiety diagnosis at baseline). For this purpose, we subtracted TL and AA at the follow-up from their respective measures at baseline to generate their deltas. We found that positive anxiety diagnostic status at baseline, compared with no diagnosis, was not associated with changes (deltas) in TL (B = 0.123, t(36) = 0.280, p = 0.781) nor with changes in AA (B = 0.897, t(43) = 0.344, p = 0.733). This reveals that by observing only diagnostic status at baseline without considering different courses and data structure (repeated measures), different conclusions can be made while observing anxiety and cellular aging markers.

Figure S1 – Unadjusted and adjusted interaction of anxiety diagnostic course on telomere length change between baseline and follow-up.


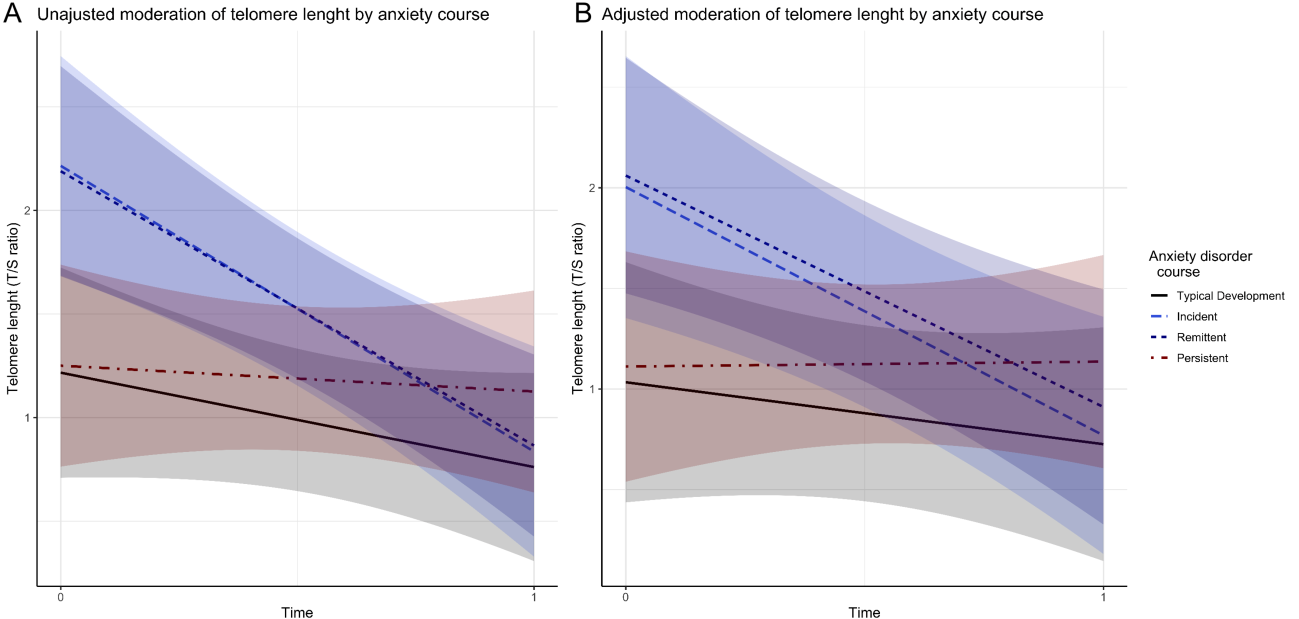


Legend: Unadjusted (A) and adjusted (B) interaction of anxiety diagnostic course (colored lines with shaded 95% CI) on telomere length change (y-axis) between baseline and follow-up five years later (x-axis).

Figure S2 – Unadjusted and adjusted interaction of anxiety diagnostic course on epigenetic age acceleration change between baseline and follow-up.


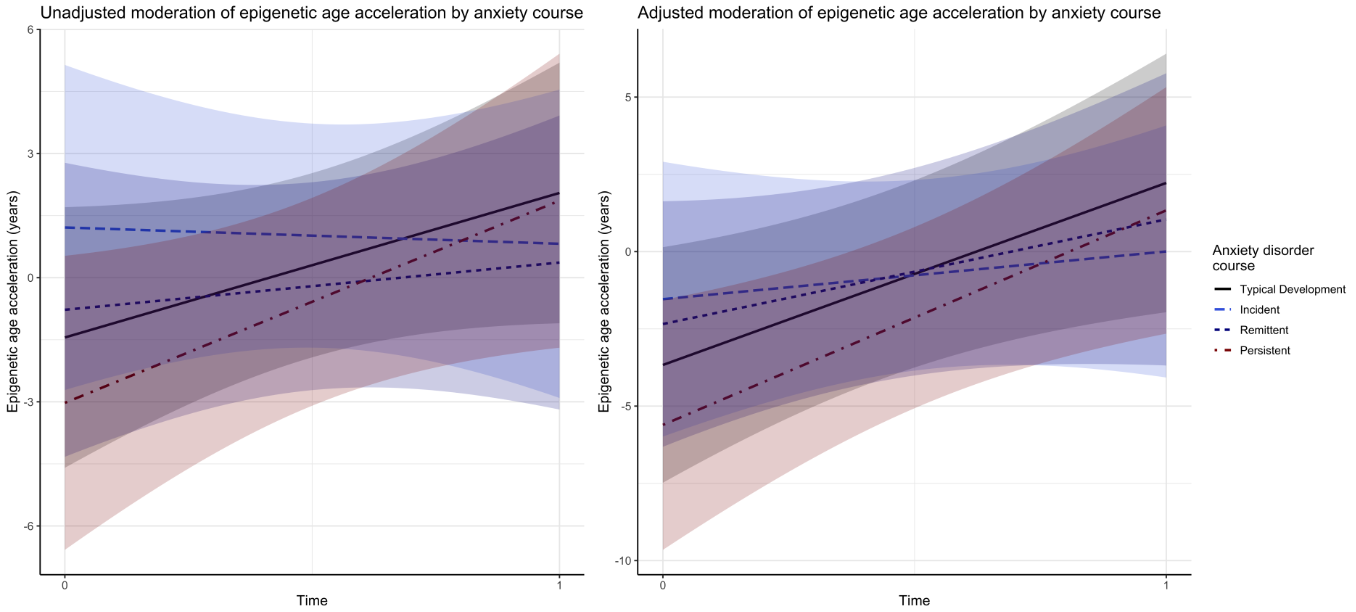


Legend: Unadjusted (A) and adjusted (B) interaction of anxiety diagnostic course (colored lines with shaded 95% CI) on epigenetic age acceleration change (y-axis) between baseline and follow-up five years later (x-axis).

| **Table S3.** Descriptive data on four adolescent anxiety diagnostic course evaluated in 2008 and 2013 | | | | | | | | | |
| --- | --- | --- | --- | --- | --- | --- | --- | --- | --- |
|  | **Baseline** | | | |  | **5-years follow-up** | | | |
| **Variables** | **Typical Development** | **Incident** | **Remittent** | **Persistent** |  | **Typical Development** | **Incident** | **Remittent** | **Persistent** |
|  | (n=18) | (n=16) | (n=22) | (n=20) |  | (n=18) | (n=16) | (n=22) | (n=20) |
| **Telomere lenght (base pairs)** | |  |  |  |  |  |  |  |  |
| Mean (SD) | 1.22 (0.566) | 2.22 (1.66) | 2.19 (1.36) | 1.25 (0.826) |  | 0.759 (0.359) ** | 0.839 (0.487) ** | 0.863 (0.732) ** | 1.13 (0.528) |
| Median [Min, Max] | 1.18 [0.519, 2.33] | 2.13 [0.557, 5.94] | 1.84 [0.576, 4.87] | 1.05 [0.566, 3.80] |  | 0.638 [0.338, 1.57] | 0.785 [0.0929, 1.82] | 0.627 [0.261, 3.39] | 1.02 [0.466, 2.27] |
| **Epigenetic age acceleration (years)** |  |  |  |  |  |  |  |  |  |
| Mean (SD) | -1.45 (7.98) | 1.21 (11.1) | -0.776 (4.80) | -3.03 (3.86) |  | 2.05 (4.14) | 0.819 (3.48) | 0.365 (4.27) | 1.86 (4.51) ** |
| Median [Min, Max] | -3.91 [-6.71, 25.4] | -1.91 [-5.82, 29.9] | -2.74 [-7.55, 9.65] | -3.66 [-8.52, 3.38] |  | 1.02 [-2.36, 10.9] | -0.0536 [-2.66, 6.33] | 0.817 [-8.78, 5.51] | 2.28 [-3.44, 11.5] |
| **Age (years)** |  |  |  |  |  |  |  |  |  |
| Mean (SD) | 13.2 (2.42) | 12.8 (2.31) | 14.6 (2.34) | 12.5 (1.95) |  | 17.2 (2.50) ** | 16.6 (2.19) ** | 18.5 (2.56) ** | 16.3 (2.08) ** |
| **Sex** |  |  |  |  |  |  |  |  |  |
| Female | 9 (50 %) | 6 (38 %) | 16 (73 %) | 15 (75 %) |  | 9 (50 %) | 6 (38 %) | 16 (73 %) | 15 (75 %) |
| Male | 9 (50 %) | 10 (62 %) | 6 (27 %) | 5 (25 %) |  | 9 (50 %) | 10 (62 %) | 6 (27 %) | 5 (25 %) |
| **Race/Ethnicity** |  |  |  |  |  |  |  |  |  |
| Caucasian | 10 (56 %) | 11 (69 %) | 14 (64 %) | 13 (65 %) |  | 10 (56 %) | 11 (69 %) | 14 (64 %) | 13 (65 %) |
| African Brazilian | 2 (11 %) | 2 (12 %) | 3 (14 %) | 4 (20 %) |  | 2 (11 %) | 2 (12 %) | 3 (14 %) | 4 (20 %) |
| Mixed | 5 (28 %) | 3 (19 %) | 5 (23 %) | 3 (15 %) |  | 5 (28 %) | 3 (19 %) | 5 (23 %) | 3 (15 %) |
| Note: Chi-squared test was applied for testing sex and Race/ethnicity between anxiety groups. Analysis of variance and Tukey post-hoc tests were used to examine telomere length, epigenetic age acceleration and chronological age differences among anxiety groups within each year and for overall sample between years (a). *, p < 0.05 compared with all other anxiety groups within the same time point (Tukey post-hoc test); **, p < 0.05 compared with the same group at baseline (t-test). | | | | | | | | | |

| Table S4 - Baseline and longitudinal analysis of relative telomere length in three different courses of anxiety disorders compared to TDC group, using generalized linear mixed regression models | | | | |
| --- | --- | --- | --- | --- |
|  | Predictor | B^a^ | p | CI 95% |
| Model 1^b^ | Incident AD | 0.998 ** | **0.009** | 0.289 – 1.708 |
|  | Remittent AD | 0.973 ** | **0.009** | 0.279 – 1.667 |
|  | Persistent AD | 0.034 | 0.924 | -0.647 – 0.715 |
|  | Time | -0.455 | 0.188 | -1.118 – 0.207 |
|  | Incident AD x Time | -0.924 | 0.073 | -1.897 – 0.049 |
|  | Remittent AD x Time | -0.869 | 0.077 | -1.799 – 0.061 |
|  | Persistent AD x Time | 0.331 | 0.498 | -0.611 – 1.273 |
| Model 2^c^ | Incident AD | 0.968 * | **0.012** | 0.262 – 1.673 |
|  | Remittent AD | 1.019 ** | **0.009** | 0.306 – 1.731 |
|  | Persistent AD | 0.077 | 0.834 | -0.613 – 0.767 |
|  | Time | -0.312 | 0.441 | -1.079 – 0.454 |
|  | Incident AD x Time | -0.918 | 0.084 | -1.903 – 0.067 |
|  | Remittent AD x Time | -0.831 | 0.102 | -1.777 – 0.115 |
|  | Persistent AD x Time | 0.337 | 0.504 | -0.615 – 1.288 |
| TDC: Typically Developing Comparisons. CI: Confidence Interval.  a Units of telomere length (T/S ratio); b Non-adjusted; c Adjusted for age, sex and ethnicity. *, p < 0.05 ; **, p < 0.01. | | | | |

| Table S5 - Baseline and longitudinal analysis of epigenetic age acceleration in three different courses of anxiety disorders compared to TDC group, using generalized linear mixed regression models | | | | |
| --- | --- | --- | --- | --- |
|  | Predictor | B^a^ | p | CI 95% |
| Model 1^b^ | Incident AD | 2.659 | 0.299 | -2.218 – 7.536 |
|  | Remittent AD | 0.669 | 0.780 | -3.930 – 5.269 |
|  | Persistent AD | -1.580 | 0.512 | -6.179 – 3.020 |
|  | Time | 3.493 | 0.126 | -0.825 – 7.811 |
|  | Incident AD x Time | -3.887 | 0.276 | -10.683 – 2.909 |
|  | Remittent AD x Time | -2.351 | 0.490 | -8.861 – 4.158 |
|  | Persistent AD x Time | 1.393 | 0.682 | -5.116 – 7.902 |
| Model 2^c^ | Incident AD | 2.129 | 0.422 | -2.813 – 7.072 |
|  | Remittent AD | 1.325 | 0.592 | -3.284 – 5.933 |
|  | Persistent AD | -1.940 | 0.430 | -6.515 – 2.634 |
|  | Time | 5.890 * | 0.031 | 0.948 – 10.833 |
|  | Incident AD x Time | -4.347 | 0.228 | -11.024 – 2.330 |
|  | Remittent AD x Time | -2.503 | 0.466 | -8.902 – 3.895 |
|  | Persistent AD x Time | 1.052 | 0.759 | -5.349 – 7.454 |
| TDC: Typically Developing Comparisons. CI: Confidence Interval.  a Units of epigenetic age acceleration; b Non-adjusted; c Adjusted for age, sex and ethnicity. *, p < 0.05 . | | | | |
